# Uso de Smartwatch na Identificação do Bloqueio Atrioventricular no Pós-Operatório de Cirurgia Cardíaca: Para Além da Detecção da Fibrilação Atrial

**DOI:** 10.36660/abc.20240131

**Published:** 2024-08-21

**Authors:** Matheus S. Moitinho, Camila R. Moreno, Rosangela Monteiro, Guilherme C. M. Rabello, Bruna M. Mariano, Pietro C. C. O. Martins, Flávio Tarasoutchi, Nelson Samesima, Alexandre de M. Soeiro, Fabio B. Jatene

**Affiliations:** 1 Instituto do Coração do Hospital das Clínicas da Faculdade de Medicina da Universidade de São Paulo São Paulo SP Brasil Núcleo de Inovação (InovaInCor) – Instituto do Coração do Hospital das Clínicas da Faculdade de Medicina da Universidade de São Paulo, São Paulo, SP – Brasil; 2 Instituto do Coração do Hospital das Clínicas da Faculdade de Medicina da Universidade de São Paulo Unidade Clínica de Valvulopatias São Paulo SP Brasil Unidade Clínica de Valvulopatias – Instituto do Coração do Hospital das Clínicas da Faculdade de Medicina da Universidade de São Paulo, São Paulo, SP – Brasil; 3 Instituto do Coração do Hospital das Clínicas da Faculdade de Medicina da Universidade de São Paulo Unidade de Eletrocardiografia São Paulo SP Brasil Unidade de Eletrocardiografia – Instituto do Coração do Hospital das Clínicas da Faculdade de Medicina da Universidade de São Paulo, São Paulo, SP – Brasil; 4 Instituto do Coração do Hospital das Clínicas da Faculdade de Medicina da Universidade de São Paulo Unidade de Emergência São Paulo SP Brasil Unidade de Emergência – Instituto do Coração do Hospital das Clínicas da Faculdade de Medicina da Universidade de São Paulo, São Paulo, SP – Brasil

**Keywords:** Saúde, Procedimentos Cirúrgicos Cardíacos, Bloqueio Atrioventricular

## Introdução

Nos cuidados cardíacos pós-operatórios, a detecção precoce e o manejo das complicações são fundamentais para a obtenção de resultados ideais. O bloqueio atrioventricular (AV) de terceiro grau, também conhecido como bloqueio atrioventricular total (BAVT), é uma preocupação significativa, que leva a sintomas como fadiga, intolerância ao esforço e eventos potencialmente fatais, como síncope ou insuficiência cardíaca,^1^ frequentemente exigindo implantação de marca-passo cardíaco (MC) para uma intervenção oportuna. No pós-operatório de cirurgia cardíaca, problemas de condução, decorrentes de alterações metabólicas ou inflamatórias, são comuns, frequentemente exigindo a colocação de MC temporários ou permanentes para estabilização dos pacientes ou sintomas. Aproximadamente 6% dos pacientes de cirurgia valvar podem precisar de MC permanente devido ao BAVT, que pode surgir no pós-operatório.^2^

A tecnologia médica, incluindo dispositivos inteligentes e plataformas de telemonitoramento remoto, tem revolucionado a assistência pós-operatória, oferecendo informações em tempo real para um monitoramento rigoroso e deteção precoce de anomalias.^3^

Os smartwatches utilizam sensores de fotopletismograma (PPG) para monitorar os batimentos e o ritmo cardíaco, analisando a atividade elétrica do coração por meio de um eletrocardiograma (ECG) de derivação única.^4^ Alguns dispositivos incluem Notificação de Ritmo Cardíaco Irregular (IHRN) para ritmos cardíacos irregulares, especialmente para fibrilação atrial.^5^

Este estudo de caso destaca a sinergia entre o conhecimento médico e a tecnologia na identificação e tratamento de problemas de condução cardíaca pós-operatória, conforme exemplificados pela identificação inovadora de um bloqueio AV de terceiro grau. A integração de dispositivos inteligentes e o monitoramento remoto desempenham um papel fundamental no tratamento das complicações do BAVT. Apresentamos um caso pós troca valvar aórtica, em que um bloqueio AV de terceiro grau assintomático foi detectado tardiamente por meio de um smartwatch, mostrando o potencial da tecnologia na melhoria dos cuidados pós-operatórios e na segurança do paciente.

### Apresentação do caso

Paciente do sexo feminino, 52 anos de idade, participante de uma pesquisa sobre monitoramento remoto usando o Smartwatch Samsung Galaxy watch 5, apresenta histórico médico incluindo hipertensão arterial sistêmica, tabagismo e consumo ativo de álcool. Foi submetida a intervenção cirúrgica aos 39 anos, optando pela substituição da valva aórtica por insuficiência aórtica significativa proveniente de valva aórtica nativa bicúspide.

Posteriormente, optou-se pela cirurgia eletiva para substituição da valva aórtica por prótese mecânica devido ao mau funcionamento da prótese biológica anterior. A substituição mecânica da valva aórtica foi bem-sucedida, com episódio de bloqueio AV transitório pós-circulação extracorpórea (CEC). Foi necessário suporte temporário de MC no pós-operatório imediato, solucionando o bloqueio AV. Sua recuperação na unidade de terapia intensiva (UTI) evoluiu satisfatoriamente.

Após a alta hospitalar, a paciente não apresentou intercorrências ou complicações, com recuperação dentro do esperado.

**Figura 1:**
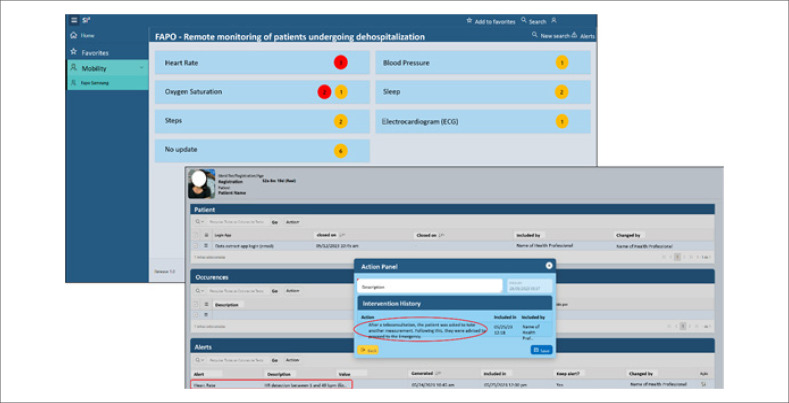
Telemonitoramento pós-substituição de válvula: plataforma alertando um evento de bradicardia. Captura de imagem da plataforma de telemonitoramento utilizada no pós-operatório para identificação de fibrilação atrial, revelando evento de bradicardia. Nota: A plataforma original está inteiramente em português brasileiro. Para fins de publicação, realizou-se uma edição de imagens para traduzir para o inglês e anonimizar a identidade da paciente e da equipe de saúde responsável pelo seu atendimento.

**Figura 2:**
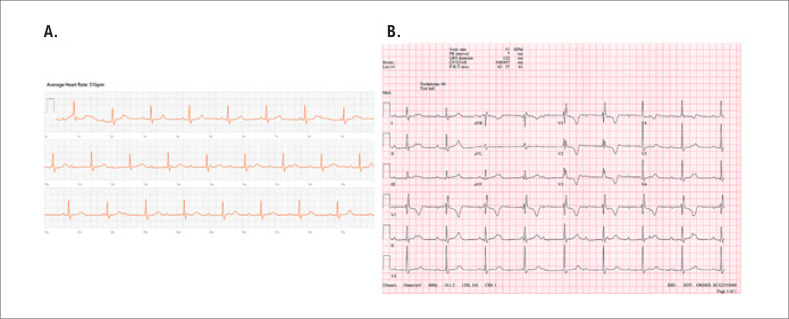
Telemonitoramento pós-substituição de válvula: ECG do smartwatch em comparação com o ECG padrão no pronto-socorro. A. Eletrocardiograma gerado pelo smartwatch durante o período de telemonitoramento do protocolo para identificação de fibrilação atrial pós-operatória, revelando traço de bloqueio atrioventricular completo. B. Eletrocardiograma obtido no pronto-socorro após encaminhamento do paciente devido à detecção de bloqueio AV no smartwatch durante o período de telemonitoramento.

Este caso ilustra a intrincada conexão entre a recuperação pós-operatória e tecnologias avançadas de monitoramento. Utilizando um smartwatch como canal de coleta de dados, duas semanas após a cirurgia, os dados do dispositivo revelaram flutuações na frequência cardíaca (Figura 1), exigindo ações clínicas subsequentes. Apesar da ausência de queixas ou distúrbios do paciente durante as teleconsultas, um exame de ECG de derivação única foi solicitado, que revelou uma frequência cardíaca de 51 batimentos por minuto. Assim, os eletrocardiogramas gerados pelo smartwatch, embora breves, mostraram claramente uma dissociação entre as ondas P e os complexos QRS (Figura 2a). Os alertas evolutivos, aliados ao discernimento da equipe de enfermagem sobre as peculiaridades do eletrocardiograma, levaram a suspeita diagnóstica de BAVT. Medidas imediatas foram tomadas para garantir o bem-estar da paciente e a comunicação facilitou sua rápida visita ao pronto-socorro, onde exames eletrocardiográficos confirmaram o diagnóstico (Figura 2b).

**Figura 3:**
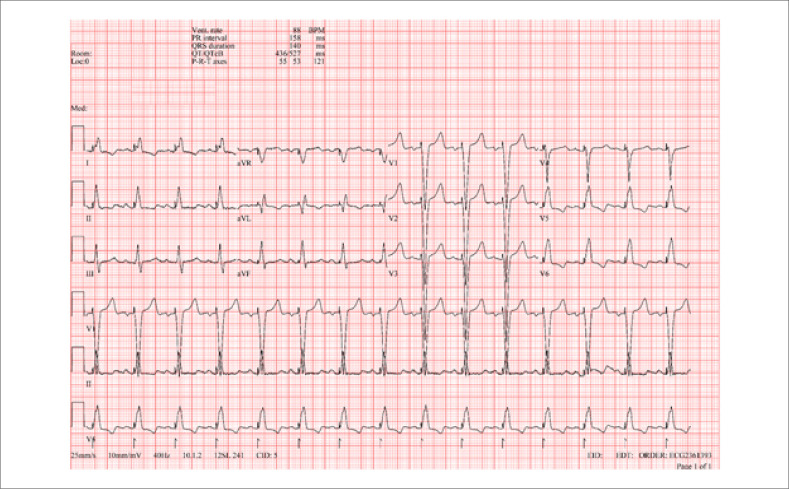
Implantação de marca-passo: pós-procedimento de ECG para BAVT. Eletrocardiograma realizado pós-implante de marca-passo definitivo por bloqueio atrioventricular total.

A identificação deste evento foi fundamental para a redefinição terapêutica, levando a uma modificação abrangente no plano de tratamento, com a implantação urgente de um marca-passo cardíaco definitivo (Figura 3).

**Figura 4:**
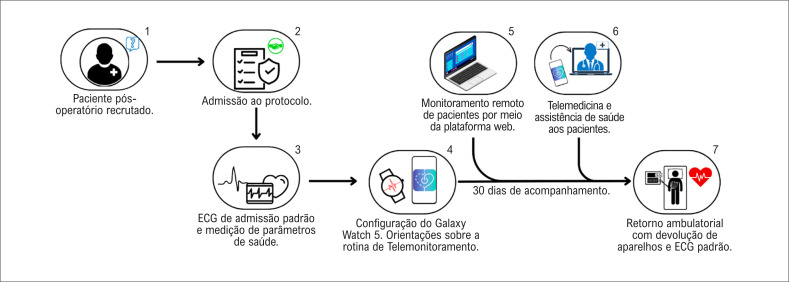
Representação esquemática da jornada de telemonitoramento do paciente. 1–2) Pacientes recrutados e admissão no protocolo. 3) Aquisição padrão de ECG e parâmetros de saúde. 4) Implantação e configuração do smartwatch e do smartphone do paciente. 5-6) Telemonitoramento remoto e assistente clínico por 30 dias. 7) Fim do protocolo.

A Figura 4 mostra uma representação esquemática da jornada da paciente no protocolo de telemonitoramento remoto. Este relato de caso recebeu aprovação ética do Comitê de Ética sob os protocolos SDC 5.874.032 e CAAE 66520122.0.0000.0068.

## Discussão

O caso ressalta o impacto transformador da integração de dispositivos inteligentes e telemonitoramento remoto aos cuidados pós-operatórios, utilizando especificamente um smartwatch para monitoramento contínuo. Esta abordagem é crucial para a detecção precoce de anomalias cardíacas, demonstrando o potencial de revolução das práticas de saúde.

O uso popular de smartwatches e tecnologias vestíveis em contextos cotidianos é um fenômeno atual. A relação simbiótica entre essas tecnologias e a saúde tem se tornado cada vez mais comum e significativa. Embora ainda em fase inicial para a prática clínica, a potencial aplicação destas tecnologias como ferramentas de apoio às decisões médicas tem sido fundamentada por evidências científicas atuais.^6^ Elas podem melhorar os cuidados ao paciente, permitindo a medição fácil e portátil de diversos parâmetros de saúde.

A utilização de smartwatches em conjunto com plataformas de telemonitoramento digital remoto representa uma realidade promissora na medicina moderna, permitindo o monitoramento contínuo e em tempo real de parâmetros essenciais de saúde, ultrapassando diversas barreiras, principalmente aquelas relacionadas com a inacessibilidade ou monitoramento contínuo.

Um ponto forte essencial deste sistema integrado reside no monitoramento meticuloso de parâmetros vitais, como frequência cardíaca, pressão arterial, saturação de oxigênio e ECG. Esse monitoramento abrangente oferece informações em tempo real sobre a dinâmica fisiológica dos pacientes, permitindo que os profissionais de saúde identifiquem rapidamente quaisquer desvios da normalidade com praticidade e confiabilidade. A natureza proativa da tecnologia permite intervenções clínicas imediatas, alterando significativamente a dinâmica da assistência pós-operatória.

O caso apresentado destaca técnicas avançadas de monitoramento, que agilizam a tomada de decisões clínicas, conforme demonstrado pela pronta resposta às anomalias cardíacas identificadas. Isto melhora a capacidade dos prestadores de cuidados de saúde em intervir rapidamente, aumentando a eficácia do tratamento, minimizando potenciais complicações e, em última análise, otimizando os resultados dos pacientes.

Um aspecto notável é a transição das avaliações intermitentes para a vigilância contínua, superando o modelo convencional de acompanhamento dos pacientes. A vigilância proativa por meio de dispositivos inteligentes e monitoramento remoto permite a deteção precoce de alterações sutis, aliada à experiência clínica, e facilitando intervenções precisas para uma jornada de recuperação mais segura e eficiente.

## Conclusão

Este caso ilustra a evolução do cenário dos cuidados pós-operatórios, enfatizando a sinergia entre dispositivos inteligentes e o telemonitoramento remoto. Isso aumenta a precisão do diagnóstico, agiliza a tomada de decisões e transforma a abordagem de acompanhamento do paciente. A adoção desses avanços tecnológicos capacita os profissionais de saúde para navegar pelas complexidades pós-operatórias, garantindo uma abordagem proativa e personalizada, que otimiza os resultados de saúde dos pacientes.
