# Supplementary material for: Uso de Smartwatch na Identificação do Bloqueio Atrioventricular no Pós-Operatório de Cirurgia Cardíaca: Para Além da Detecção da Fibrilação Atrial
Source: Arq Bras Cardiol. 2024 Aug 21;121(8):e20240131. [Article in Portuguese] doi: 10.36660/abc.20240131 (PMC11516154; doi:10.36660/abc.20240131)
Supplement: Translation

# Translation: Smartwatch for Identification of Atrioventricular Block in Post-cardiac Surgery: Going Beyond Atrial Fibrillation Detection

**DOI:** 10.36660/abc.20240131i

**Authors:** Matheus S. Moitinho, Camila R. Moreno, Rosangela Monteiro, Guilherme C. M. Rabello, Bruna M. Mariano, Pietro C. C. O. Martins, Flávio Tarasoutchi, Nelson Samesima, Alexandre de M. Soeiro, Fabio B. Jatene

**Affiliations:** 1 São Paulo SP Brazil Núcleo de Inovação (InovaInCor) – Instituto do Coração do Hospital das Clínicas da Faculdade de Medicina da Universidade de São Paulo, São Paulo, SP – Brazil; 2 São Paulo SP Brazil Unidade Clínica de Valvulopatias – Instituto do Coração do Hospital das Clínicas da Faculdade de Medicina da Universidade de São Paulo, São Paulo, SP – Brazil; 3 São Paulo SP Brazil Unidade de Eletrocardiografia – Instituto do Coração do Hospital das Clínicas da Faculdade de Medicina da Universidade de São Paulo, São Paulo, SP – Brazil; 4 São Paulo SP Brazil Unidade de Emergência – Instituto do Coração do Hospital das Clínicas da Faculdade de Medicina da Universidade de São Paulo, São Paulo, SP – Brazil

**Keywords:** Health, Cardiac Surgical Procedures, Atrioventricular Block

## Introduction

In postoperative cardiac care, early detection and management of complications are crucial for optimal outcomes. Third-degree atrioventricular (AV) block, also known as complete heart block (CHB), is a significant concern, leading to symptoms like fatigue, exertion intolerance, and life-threatening events such as syncope or heart failure,^1^ often necessitating cardiac pacemaker (CP) implantation for timely intervention. In the postoperative period of cardiac surgery, conduction issues stemming from metabolic or inflammatory changes are common, necessitating temporary or permanent CPs to stabilize patients or symptoms. Approximately 6% of valve surgery patients may require a permanent CP due to CHB, which can emerge post-surgery.^2^

Medical technology, including smart devices and remote telemonitoring platforms, has revolutionized postoperative care, offering real-time insights for close monitoring and early anomaly detection.^3^

Smartwatches utilize photoplethysmography (PPG) sensors to monitor heartbeat and rhythm, analyzing the heart's electrical activity through a single-lead electrocardiogram (ECG).^4^ Some devices include Irregular Heart Rhythm Notification (IHRN) for irregular heart rhythms, particularly for atrial fibrillation.^5^

This case study highlights the synergy between medical knowledge and technology in identifying and managing postoperative cardiac conduction problems, exemplified by the innovative identification of a third-degree AV block. Integration of smart devices and remote monitoring plays a pivotal role in addressing CHB complications. We present a case after aortic valve replacement, where an asymptomatic third-degree AV block was late detected through a smartwatch, show technology's potential in enhancing postoperative care and patient safety.

### Case presentation

A 52-year-old female patient, part of a research study on remote monitoring using the Samsung Galaxy 5 Smartwatch, has a medical history that includes systemic hypertension, smoking, and active alcohol consumption. She underwent surgical intervention at 39 years old, opting for aortic valve replacement due to significant aortic insufficiency from a bicuspid native aortic valve.

Later, due to the malfunction of the previous biological prosthesis, the patient chose elective surgery to replace her aortic valve with a mechanical prosthesis. The mechanical aortic valve replacement was successful, with a transient AV block episode post-cardiopulmonary bypass (CPB). Temporary pacemaker support was necessary during the immediate postoperative period to resolve the AV block. Her recovery in the intensive care unit (ICU) progressed satisfactorily.

Post-hospital discharge, the patient exhibited no events or complications, with recovery within the expected range.

This case illustrates the intricate connection between postoperative recovery and advanced monitoring technologies. Utilizing a smartwatch as the data collection conduit, two weeks post-surgery, device data revealed heart rate fluctuations (Figure 1), prompting subsequent clinical actions. Despite the absence of patient complaints or disturbances during teleconsultations, a single-lead ECG test was ordered, uncovering a heart rate of 51 beats per minute. Crucially, the smartwatch-generated electrocardiograms, though brief, clearly showed a dissociation between the P waves and QRS complexes (Figure 2a). The evolving alerts, combined with the nursing team's discernment of electrocardiogram peculiarities, led to the presumed diagnosis of CHB. Prompt measures were taken to ensure the patient's well-being, and communication facilitated her swift visit to the emergency department, where electrocardiographic examinations confirmed the diagnosis (Figure 2b).

**Figure 1:** Telemonitoring Post-Valve Replacement: Platform Alerting a Bradycardia Event. Image capture from the telemonitoring platform used during the postoperative period to identify atrial fibrillation, revealing a bradycardia event. Note: The original platform is entirely in Brazilian Portuguese. The image was edited for publication to translate it into English and to anonymize the identities of the patient and the healthcare team responsible for her care.

**Figure 2:** Telemonitoring Post-Valve Replacement: Smartwatch ECG vs. ECG Standard in the Emergency Department. A. Electrocardiogram generated by the smartwatch during the telemonitoring period of the protocol for the identification of postoperative atrial fibrillation, revealing a trace of complete atrioventricular block. B. Electrocardiogram obtained in the emergency department after the patient was referred following the detection of an AV block on the smartwatch during the telemonitored period.

The identification of this event was pivotal for therapeutic redefinition, leading to a comprehensive modification in the treatment plan, necessitating the urgent implantation of a definitive cardiac pacemaker (Figure 3).

**Figure 3:** Pacemaker Implantation: ECG Post-Procedure for CHB. Electrocardiogram conducted post-implantation of a permanent pacemaker due to a complete atrioventricular block.

Figure 4 provides a schematic representation of the patient's journey in the remote telemonitoring protocol. This case report received ethical approval from the Ethics Committee under protocol numbers SDC 5.874.032 and CAAE 66520122.0.0000.0068.

**Figure 4:** Figure of the patient's telemonitoring journey. 1–2) Patients recruited and admitted to the protocol. 3) Standard ECG and health parameters acquisition. 4) Patient's smartwatch and smartphone deployment and setup. 5-6) Remote telemonitoring and clinical assistance for 30 days. 7) Protocol's end.

## Discussion

The case underscores the transformative impact of integrating smart devices and remote telemonitoring into postoperative care, specifically utilizing a smartwatch for continuous monitoring. This approach is crucial for the early detection of cardiac anomalies, demonstrating the potential to revolutionize healthcare practices.

The popular use of smartwatches and wearable technologies in everyday contexts is a current phenomenon. The symbiotic relationship between these technologies and health has become increasingly common and significant. Although still in its early stages for clinical practice, the potential application of these technologies as tools to support clinical decisions is being substantiated by current scientific evidence.^6^ They can enhance care by enabling easy and portable measurement of various health parameters.

The use of smartwatches in conjunction with remote digital telemonitoring platforms represents a promising reality in modern medicine, allowing continuous and real-time monitoring of essential health parameters, overcoming various barriers, particularly those related to inaccessibility or continuous monitoring.

An essential strength of this integrated system lies in its meticulous tracking of vital parameters such as heart rate, blood pressure, oxygen saturation, and ECG. This comprehensive monitoring provides real-time insights into patients’ physiological dynamics, enabling healthcare professionals to swiftly identify deviations from the norm with practicality and reliability. The proactive nature allows for prompt clinical interventions, significantly changing postoperative care dynamics.

The case highlights advanced monitoring techniques expediting clinical decision-making, demonstrated by the prompt response to identified cardiac anomalies. This improves healthcare providers’ capacity to intervene swiftly, enhancing treatment effectiveness and minimizing potential complications, ultimately optimizing patient outcomes.

A notable aspect is the transition from intermittent assessments to continuous surveillance, surpassing the conventional patient follow-up model. Proactive vigilance through smart devices and remote monitoring enables early detection of subtle changes, coupled with clinical expertise, facilitating precise interventions for a safer and more efficient recovery journey.

## Conclusion

In conclusion, the case illustrates the evolving postoperative care landscape, emphasizing the synergy between smart devices and remote telemonitoring. This enhances diagnostic accuracy, expedites decision-making, and transforms the patient follow-up approach. Embracing these advancements equips healthcare providers to navigate postoperative complexities, ensuring a proactive, personalized approach that optimizes patient outcomes in modern healthcare.
